# Book Notices

**Published:** 1869-01-01

**Authors:** 


### *BOOK NOTICES.*

De la Cantharide Officinale These de Pharmacie: Prísentú et soutenue a l'école superieure de pharmacie, par Armand Fumonze, Docteur en Médecine, Pharmacien de 1re. classe, Membre de la société entomologique de France et de la société Chimique de Paris. Paris: Chez Germer Balliere Libraire-Editeur Rue de L'Ecole-de-Médecine, 17.

The above is the title of a most comprehensive monograph, another one of those many monuments of the patient industry, minute investigation, accurate observation of our Gallic confrêres, with which the field of science is adorned.

The author divides his work into four chapters, in the first of which he treats of the natural history of Officinal Cantharides, in the second, of its chemical history, in the third, of the causes which may change or impair its properties, and in the fourth, of the insects and acari which are found intermingled with it.

According to our author, Hippocrates recommended the use of this drug internally in Jaundice, Apoplexy and Dropsy, but was ignorant of its vesicating properties, which seem to have been discovered in the first century by Archigenus, or according to some by Areteus. It appears to have been subsequently used to protect textile fabrics from the ravages of moths, and somewhat later, as an aphrodisiac, both of which properties our author refuses to accord to it.

After commenting upon the confusion existing amongst the older writers about the identity of the insect, and comparison of the writings of Linnæus, Fabricus, Geoffrey de Geer, Latreille, Dumeril, Andouin and Mulsant, from which it appears that the insect possessing vesicating properties, belongs to the family (tribu) *Meloides*, which is divided into three groupes *Meloe*, *Mylabris* and *Cantharis*, of these the *Mylabris chiconiè* is used in Hindostan, as is likewise the *Meloe trianthemæ*, (which is found upon the blossom of the Cucurbitaceæ), *Mylabris cyanescens* and *Mylabris variabiles.* The *Cantharis vitata* (from the potatoe blossom), is used in South America, together with the *Cantharis adspersa.* The latter of which, according to M. Courbon, possesses very energetic vesicating properties without any irritant action upon the genito-urinary organs. The *Meloe proscaraæus* is used in Spain in Veterinary medicine.

Assuming the *Cantharis vesicatoria* of Linnæus as the officinal species, the author supplies us with a most graphic description of the insect, illustrated with excellent lithographic plates.

In the second chapter, the author reviews in detail the chemical history of Cantharides, from the earliest abortive attempts of various experimenters, previous to, and during the seventeenth century, to detect by analysis its active principle, together with the first apparently systematic one of Moyse Charas, Paris 1681, who seems to have anticipated, almost completely, the more elaborate one of Thouvenel in 1778, which contained essentially the same elements, and appears to have almost completely anticipated that of Beaupoil in 1803.

The first analysis which was successful in indicating the active principle to which the vesicating properties of the insect are due, was that of Robiquet in 1810, and of which the author gives us the detail, and which constitutes what the author terms the last term of the first period of the chemical history of Cantharides.

The second period seems to have commenced with investigations into the method of isolating this active principle, the oldest of which was that of Thierry in 1835, who made use of Alcohol and Ether as menstrua. Our author objects to both of these, to the first from its capacity for dissolving cêrtain other constituent principles, and to the second as not being more active as a solvent than the first, and at the same time dangerous from its inflammability. He prefers greatly, as a menstruum, Chloroform, proposed by Proctor ten years ago, to be succeeded by solution in Sulphuret of Carbon which dissolves every thing except the Cantharidine, which it precipitates. This modification of the process of Proctor is claimed by the author as originating with himself, and he presents us with a *resume* of three experiments illustrative of the process.

In the third chapter, in discussing the causes which may diminish or alter the properties of Cantharides, one of the first referred to, is excessive heat in the drying process hence the open air is preferred, as a medium, to stove heat, and a temperature not exceeding 100°, an elevation to 120° or 125° having been found to diminish materially the activity of the product.

The process of drying in the open air renders the Cantharides liable to the attacks of insects who deposit their larvæ therein. Cantharides are also adulterated by the addition of the powder of the Euphorbium; by the addition of non-vesicating insects; by plunging in oil, to add to their weight, and by the addition of insects exhausted of their active principle

To avoid all sophistications aud adulterations, the author prescribes a very simple rule: select entire, unbroken insects, dry and having the characteristic colors and odor.

With regard to the parasites preying upon these insects and their effects, the author has determined by experiment that — 1st. The Cantharidine (the active principle) is not destroyed by these animals.

2nd. That old Cantharides, although yielding to analysis no Cantharidine, may still preserve all their vesicating properties.

3rd. That moisture being one of the most energetic causes of their degeneration, they should be preserved in a dry place.

This interesting monogram terminates with a detailed description of the insects and acari, which are found mingled with Cantharides either fortuitously or as parasites. This description is accompanied by excellent lithographic illustrations. To the practical pharmacist, or the country practitioner compelled by the exigencies of his position to rely upon his own judgment, in the choice of the agents to be employed in his practice, this monogram can not fail to prove both interesting and valuable.

W. H.

Annual Report of the Surgeon General, United States Army, 1868.

The report gives us the annual financial statement of the department; statistics of sickness, wounded, and the mortality of the white and colored troops, respectively, in their various localities; and the present stage of progress of the Medical and Surgical History of the late war; the condition of the Army Museum, etc., etc. At the date of the report there were forty-nine vacancies in the office of Assistant Surgeon.

A Treatise on Physiology and Hygiene; for schools, families and colleges. By J. C. Dalton, M.D., Professor of Physiology in the College of Physicians and Surgeons, N. Y. With illustrations. New York: Harper & Brothers, publishers. London: Sampson, Low & Marston, 1868. Pp. 399.

The name of the distinguished author of this compend is a sufficient guarantee of the accuracy of the scientific information it contains. From such examination as we have been enabled to give it, it seems well adapted to the designed purpose. It is well illustrated, and provided with a full series of questions, appended to each chapter. We trust to see it favorably received by the public.

A Hand-Book of Vaccination. By Edward C. Leaton, M.D., Medical Inspector to the Privy Council. Philadelphia: J. B. Lippincott & Co., 1868. Pp. 383.

A very complete *resume* of the subject, which should be in the possession of every practitioner. It is divided into fourteen chapters, discussing natural cow-pox; horse-pox; pocks in other animals analogous to the same; vaccinia and vaccinating; lymph supply, conveyance and storage; success of the operation; insusceptibility of individuals; degeneration of lymph; protection afforded; re-vaccination; stamping out local out-breaks of variola; objections to, and alleged dangers of, the practice.

The Opium Habit; with suggestions as to the remedy. New York: Harper & Brothers, publishers, Franklin Square, 1868. Pp. 335.

An intensely interesting work, both to the professional and non-professional reader. De Quincey, Coleridge, William Blair, Robert Hall, John Randolph, William Wilberforce, and other victims of the habit are self-portrayed, together with the author's own experience.

Within our own knowledge the habit is wide-spread, prevailing and increasing. Physicians must attempt to stay its disastrous progress. Better still — *obsta principiis.*

The therapeutics need revising. Our own opinion is, that medical science can furnish better management of these unhappy cases than this book suggests. The difficulties, however, are herein forcibly set forth,

Lectures on the Study of Fever. By Alfred Hudson, M.D., M.R.I.A., Physician to the Meath Hospital. Philadelphia: Henry C. Lea, 1869. Pp. 316.

A very useful book, far in advance of many works on the subject, even of recent date. The author "furnishes the student with a guide to his bedside analysis of each case, by treating febrile phenomena in succession; first, generally or abstracted; and, secondly, in their relation to each form of the disease."

Fifteen lectures and an appendix are given — the latter being devoted to details of some illustrative cases, and the controverted question of identity or non-identity of fever poisons.

We regret that, amid so much to commend, we find it necessary to insist that some portions of his therapeutics are worthy of nothing but the severest reprehension. The day of blood-letting, emetics, and sedatives, such as tartar emetic (strongly recommended by this author), in the treatment of fever as such has gone by. Transatlantic prejudices are most forcibly illustrated on pages 208–9–10–11–12. Striking out these, and the book would infinitely better please American practitioners, who now believe in conservative, not in destructive medication. The analysis, which the author himself affords, of the principles of his treatment, is abundantly sufficient to overthrow the part referred to on the pages above noted. Why will not medical men listen to the mandates of physiological science, rather than be the blind servitors of mere traditional empiricism?

### *Books and Pamphlets Received.*

TRANSACTIONS OF THE AMERICAN DENTAL ASSOCIATION. (JOURNAL, page 614.)

This volume does great credit to the American Dental profession. It includes the proceedings at both the Chicago meeting in 1865, and that at Boston in 1866.

The welcoming address, by Dr. W. W. Allport, of this city, at the first meeting; the address on Specialties, by the late Prof. Daniel Brainard, were excellent at the time, and well worth preservation. Dr. Atkinson's report of the Committee on Pathology and Surgery, is also a paper deserving of the careful perusal of both medic l men and dentists. General surgeons will also find much of interest in Dr. McQuillen's essay on "Dental Manipulation" (p 127). Among other valuable contents, the discussion upon Dental Physiology (p. 409), and that upon Dental Education (p. 482).

The volume, as a whole, does credit to our friends of the dental specialty, and we regret that the pressure upon the pages of the JOURNAL does not now permit, what we long ago intended, copious extracts, or at least abstracts, from its many interesting and important pages.

